# A comparison of pedigree, genetic and genomic estimates of relatedness for informing pairing decisions in two critically endangered birds: Implications for conservation breeding programmes worldwide

**DOI:** 10.1111/eva.12916

**Published:** 2020-01-27

**Authors:** Stephanie J. Galla, Roger Moraga, Liz Brown, Simone Cleland, Marc P. Hoeppner, Richard F. Maloney, Anne Richardson, Lyndon Slater, Anna W. Santure, Tammy E. Steeves

**Affiliations:** ^1^ School of Biological Sciences University of Canterbury Christchurch New Zealand; ^2^ Tea Break Bioinformatics, Ltd Palmerston North New Zealand; ^3^ New Zealand Department of Conservation Twizel New Zealand; ^4^ Institute for Clinical Molecular Biology Christian‐Albrechts‐University Kiel Kiel Germany; ^5^ New Zealand Department of Conservation Christchurch New Zealand; ^6^ The Isaac Conservation and Wildlife Trust Christchurch New Zealand; ^7^ New Zealand Department of Conservation Rangiora New Zealand; ^8^ School of Biological Sciences University of Auckland Auckland New Zealand

**Keywords:** conservation breeding, conservation genetics, conservation genomics, pairing recommendations, PMx, relatedness

## Abstract

Conservation management strategies for many highly threatened species include conservation breeding to prevent extinction and enhance recovery. Pairing decisions for these conservation breeding programmes can be informed by pedigree data to minimize relatedness between individuals in an effort to avoid inbreeding, maximize diversity and maintain evolutionary potential. However, conservation breeding programmes struggle to use this approach when pedigrees are shallow or incomplete. While genetic data (i.e., microsatellites) can be used to estimate relatedness to inform pairing decisions, emerging evidence indicates this approach may lack precision in genetically depauperate species, and more effective estimates will likely be obtained from genomic data (i.e., thousands of genome‐wide single nucleotide polymorphisms, or SNPs). Here, we compare relatedness estimates and subsequent pairing decisions using pedigrees, microsatellites and SNPs from whole‐genome resequencing approaches in two critically endangered birds endemic to New Zealand: kakī/black stilt (*Himantopus novaezelandiae*) and kākāriki karaka/orange‐fronted parakeet (*Cyanoramphus malherbi*). Our findings indicate that SNPs provide more precise estimates of relatedness than microsatellites when assessing empirical parent–offspring and full sibling relationships. Further, our results show that relatedness estimates and subsequent pairing recommendations using *PMx* are most similar between pedigree‐ and SNP‐based approaches. These combined results indicate that in lieu of robust pedigrees, SNPs are an effective tool for informing pairing decisions, which has important implications for many poorly pedigreed conservation breeding programmes worldwide.

## 1 INTRODUCTION

In order to recover the world's rarest species, a multifaceted approach is needed to address the factors that cause species decline and those that promote species resilience (Grueber et al., 2019; Jamieson 2015; Soulé, 1985). A critical facet of threatened species recovery is genetic management (Frankham, 2005; O’Grady et al., 2006; Spielman, Brook, & Frankham, 2004), including conservation breeding, where individuals in intensively managed populations are paired to minimize inbreeding and maximize genetic diversity in an effort to maintain evolutionary potential (Ballou & Lacy, 1995; Ballou et al., 2010; de Villemereuil et al., 2019; Giglio, Ivy, Jones, & Latch, 2016; Ivy & Lacy, 2012). In these conservation breeding programmes, offspring may remain in captivity as an insurance population (e.g., the Tasmanian devil, *Sarcophilius harissii*, Hogg et al. 2015) or they may be translocated to the wild (e.g., California condor, *Gymnogyps californianus*; Walters et al., 2010) to enhance recovery efforts. In addition to demographic considerations (e.g. Moore, Converse, Folk, Runge, & Nesbitt, 2012; Slotta‐Bachmayr, Boegel, Kaczensky, Stauffer, & Walzer, 2004; Tenhumberg, Tyre, Shea, & Possingham, 2004), current best practice for making pairing decisions in conservation breeding programmes is to use available ancestry data from multigenerational pedigrees to estimate kinship—a metric of pairwise coancestry or relatedness—between all living individuals in a population (Ballou & Lacy, 1995; Ballou et al., 2010; Ivy & Lacy, 2012; Lacy, 1995). Individuals are paired to minimize mean kinship (i.e., average pairwise relatedness among all others in the population, including oneself), which has been shown to maximize founder representation and minimize inbreeding over time (Ballou & Lacy, 1995; Lacy, 2012; Willoughby et al., 2015).

Pedigrees are the tool of choice for estimating relatedness in conservation breeding programmes, including hundreds managed by the worldwide zoo and aquarium community (e.g., the Association of Zoos and Aquariums, or AZA; Hammerly, de la Cerda, Bailey, & Johnson, 2016; Jiménez‐Mena, Schad, Hanna, & Lacy, 2016; Long, Dorsey, & Boyle, 2011). Still, there are inherent assumptions that, when violated, hinder pedigree accuracy. For example, pedigrees assume no variance in founder relationships (i.e., all founders are equally unrelated; Ballou, 1983), which is unlikely for many highly threatened wild populations, as most have experienced one or more historical population bottlenecks and founders sourced from these remnant wild populations will have variance in relatedness values (Bergner, Jamieson, & Robertson, 2014; Hogg et al., 2019). Simulation studies suggest that when kinship‐based approaches are used for pairing, complete pedigrees with substantial depth (>5 generations recorded) are robust enough to reflect true relatedness and inbreeding estimates despite violating this assumption (Balloux, Amos, & Coulson, 2004; Pemberton, 2004; Rudnick & Lacy, 2008). However, in many conservation breeding programmes, wild founders are routinely sourced to supplement captive populations (e.g., kākāriki karaka, *Cyanoramphus malherbi*, this manuscript) and to reduce the risk associated with adaptation to captivity (Frankham, 2008). Under these circumstances, the assumption of no variance in founder relationships can be repeatedly violated, leading to significant underestimation of relatedness and inbreeding (Hogg et al., 2019). In addition to these caveats, many intensively managed populations are poorly pedigreed, meaning these pedigrees contain missing information (i.e., unknown parentage due to matings that include unidentified individuals or extra‐pair parentage; Bérénos, Ellis, Pilkington, & Pemberton, 2014; Lacy, 2009; Pemberton, 2008; Putnam & Ivy, 2014) or record‐keeping errors (e.g., Hammerly et al., 2016).

Even when pedigrees are of high depth, have no missing information, and contain no errors, expected relatedness between individuals can differ from realized relatedness, as pedigrees rely on probabilities as opposed to empirical estimates of genome sharing (Hill & Weir, 2012; Kardos, Luikart, & Allendorf, 2015; Speed & Balding, 2015; Willoughby et al., 2015). Based on Mendelian inheritance, pedigrees estimate the probability that two alleles, one chosen at random from each of two individuals, are identical by descent (IBD) from a common ancestor (Ballou, 1983; Lacy, 1995). When using a pedigree, the relatedness coefficient (*R*) for parents and offspring, as well as for full siblings, is 0.5 when inbreeding is not present, indicating each pair shares 50% of their genomic information. While parents do contribute 50% of their autosomal genomic information to their gametes, the combined effects of recombination, independent assortment and random fertilization can lead to a larger range of realized relatedness between full siblings (Hill & Weir, 2011, 2012; Speed & Balding, 2015). For example, a simulation study in humans revealed that realized relatedness between full siblings could range anywhere from 0.37 to 0.61 (Visscher et al., 2006); however, this range can vary depending on the genome architecture of the species in question (e.g., number and size of chromosomes and the frequency and location of recombination events; Hill & Weir, 2011; Kardos et al., 2015; Knief, Kempenaers, & Forstmeier, 2017).

An alternative approach for populations lacking robust pedigrees is to use genetic‐based estimates of pairwise relatedness to inform pairing decisions (Attard et al., 2016; Pemberton, 2004, 2008; Premachandra, Nguyen, & Knibb, 2019; Slate et al., 2004). This approach typically uses 8–30 microsatellite markers and empirical allele frequencies to estimate the probability that two alleles chosen at random from two individuals are IBD from a common ancestor (Speed & Balding, 2015). To date, numerous conservation breeding programmes have used a genetic approach to inform pairing recommendations, repair studbooks and resolve unknown parentage assignments, including programmes for the near‐threatened Parma wallaby (*Macropus parma*; Ivy, Miller, Lacy, & DeWoody, 2009), the vulnerable Jamaican yellow boa (*Epicrates subflavus*; Tzika, Remy, Gibson, & Milinkovitch, 2009), the critically endangered Anegada iguana (*Cyclura pinguis*; Mitchell et al., 2011) and the critically endangered Attwater's prairie‐chicken (*Tympanuchus cupido attwateri*; Hammerly et al., 2016; Hammerly, Morrow, & Johnson, 2013). While some empirical research indicates that a large and diverse panel of microsatellites produces diversity estimates that are representative of genome‐wide diversity and can be more useful than shallow pedigrees (e.g., Forstmeier, Schielzeth, Mueller, Ellegren, & Kempenaers, 2012), more recent simulation studies indicate that microsatellites provide less precision for relatedness and inbreeding, particularly in genetically depauperate endangered species where allelic diversity is low (i.e., <4 alleles per locus in the founding population; Robinson, Simmons, & Kennington, 2013; Taylor, 2015; Taylor, Kardos, Ramstad, & Allendorf, 2015). While the use of larger panels of diverse microsatellites may circumvent this issue for some species (e.g., Bergner et al., 2014; Gooley, Hogg, Belov, & Grueber, 2017), one simulation study for genetically depauperate endangered species shows that little precision is gained beyond 40 microsatellites, leading to inaccurate estimates of relatedness (Taylor et al., 2015). Recent studies argue that a better indication of genome‐wide diversity can be obtained from genomic‐based estimates of relatedness based on large numbers of genome‐wide single nucleotide polymorphisms (i.e., SNPs; Knief et al., 2015; Taylor, 2015; Taylor et al., 2015).

Given the decreasing cost of high‐throughput sequencing (Hayden, 2014) and the increasing amount of genomic resources readily available for nonmodel species (Galla et al., 2019), producing thousands of SNPs is now possible for many highly threatened species and provides an exciting opportunity for use in conservation breeding programmes (Galla et al., 2016; He, Johansson, & Heath, 2016). Indeed, there are several recent examples of genome‐wide SNPs being used for relatedness in conservation, ecology and evolution (e.g., De Fraga, Lima, Magnusson, Ferrão, & Stow, 2017; Escoda, González‐Esteban, Gómez, & Castresana, 2017), with some studies indicating that genome‐wide SNPs provide greater precision in estimating relatedness and inbreeding compared to robust pedigrees (Kardos et al., 2015; Santure et al., 2010; Wang, 2016) or microsatellites (Attard, Beheregaray, & Möller, 2018; Bérénos et al., 2014; Hellmann et al., 2016; Keller, Visscher, & Goddard, 2011; Lemopoulos et al., 2019; Li, Strandén, Tiirikka, Sevón‐Aimonen, & Kantanen, 2011; Thrasher, Butcher, Campagna, Webster, & Lovette, 2018).

To our knowledge, no study has compared pedigree‐, genetic‐ and genomic‐based approaches for estimating relatedness to inform pairing decisions for conservation breeding programmes, despite there being over 350 vertebrates worldwide that are captive bred for release to the wild (Smith et al., 2011). Here, we evaluate these three approaches using two critically endangered birds endemic birds to Aotearoa New Zealand—the kakī/black stilt (*Himantopus novaezelandiae*) and kākāriki karaka/orange‐fronted parakeet (*C. malherbi*)—as proof of concept. Kakī and kākāriki karaka are excellent candidates for this research as both have active conservation breeding programmes, as well as multigenerational pedigrees (this study), microsatellite panels (Andrews, Hale, & Steeves, 2013; Steeves, Hale, & Gemmell, 2008) and genomic resources including species‐specific reference genomes and whole‐genome resequencing data (Galla et al., 2019; this study). In addition, because captive breeding pairs for both species are housed in separate enclosures and all offspring are intensively managed, kakī and kākāriki karaka present an excellent opportunity to examine relatedness in known family groups.

**Figure 1:**
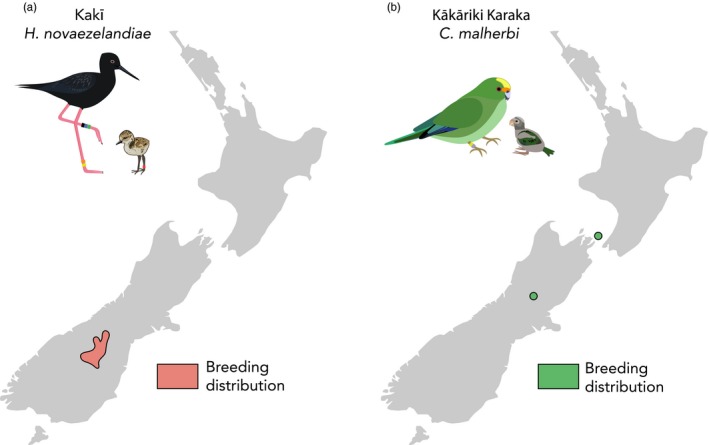
Current breeding distributions of wild kakī (a) and kākāriki karaka (b) in Aotearoa

Once found on both main islands of Aotearoa, kakī experienced significant population declines throughout the 20th century due to introduced mammalian predators (e.g., feral cats, *Felis catus*; stoats, *Mustela erminea*; and hedgehogs, *Erinaceus europaeus*) along with braided river habitat loss and degradation (Sanders & Maloney, 2002). Today, an estimated 129 kakī are largely restricted to braided rivers of Te Manahuna/The Mackenzie Basin (Department of Conservation, *personal comm.*; Figure 1a) and recovery efforts include a conservation breeding programme that was initiated in the early 1980s (Reed, 1998). In addition to breeding birds in captivity, the kakī recovery programme also collects eggs from intensively monitored wild nests and rears them in captivity before wild release. Similar to kakī, kākāriki karaka were also once found on both main islands of Aotearoa and experienced population declines in the 19th and 20th centuries due to introduced mammalian predators (e.g., brushtail possums, *Trichosurus vulpecula*; rats, *Rattus rattus* and *R. norvegicus*; and stoats) and habitat loss (Kearvell & Legault, 2017). Today, breeding populations of an estimated 100–300 kākāriki karaka are restricted to beech (*Nothofagus *spp.) forests in three North Canterbury Valleys (the Hawdon, Hurunui, and Poulter) and to Oruawairua/Blumine Island in the Marlborough Sounds (Department of Conservation, *unpublished data*; Figure 1b). Recovery efforts include a conservation breeding programme initiated in 2003, with founders sourced from the Poulter, Hawdon and Hurunui Valleys. In most instances, offspring from pairings are released to the Hurunui Valley for wild supplementation. More recently, offspring are also released into the Poulter Valley to encourage pairing with the remaining birds from an extremely small remnant wild population (Department of Conservation, *personal comm.*). Eggs from these pairs are harvested, brought into captivity and fostered under surrogate birds, with hatchlings incorporated into the conservation breeding programme. Kakī belong to the Order Charadriiformes and are relatively long‐lived braided river specialists that breed predictably within the bounds of a spring and summer season (Pierce, 2013). In contrast, kākāriki karaka belong to the Order Psittaciformes and are relatively short‐lived beech forest specialists capable of breeding year‐round, with prolific breeding periods associated with food abundance (e.g., beech forest masting events; Kearvell & Legault, 2017).

Here, we compare relatedness estimates from pedigree, microsatellites and genome‐wide SNPs using known parent–offspring and full sibling relationships. We then compare pairing recommendations among these three approaches to assess how each translates to effective conservation management. Given that kakī and kākāriki karaka represent two taxonomically distinct bird species with different life history strategies, we anticipate the results of our research may be applicable to the wider conservation breeding community.

## 2 MATERIALS AND METHODS

### 2.1 Sample collection and DNA extraction

Animal ethics approval for this project has been granted by the New Zealand Department of Conservation (i.e., DOC; permit number AEC 283). Captive kakī and kākāriki karaka are managed by DOC at two facilities in Aotearoa: the DOC Kakī Management Centre in Twizel and the Isaac Conservation and Wildlife Trust in Christchurch. Kakī used in this study are 36 individuals sampled between 2014 and 2017, including 24 individuals from six captive family groups and 12 individuals from wild parents that represent diverse lineages based on the pedigree. Kākāriki karaka sampled in this study are 36 individuals sampled between 2015 and 2019, including individuals from eight captive family groups and one wild individual from the Poulter Valley of North Canterbury (Table 1).

**Table 1:** Family group sampling strategy used in this study, based on pedigree data

Species	No. of Sampled Individuals	No. of family groups	No. of parent–offspring relationships	No. of sibling relationships
Kakī	36	6	24	7
Kākāriki Karaka	36	8	52	48

Blood, feather or tissue samples were sampled from each bird during routine health checks by DOC and Isaac Conservation and Wildlife Trust staff and immediately transferred into 95% molecular grade ethanol and stored at −80°C. High quantity and quality DNA was extracted using a lithium chloride chloroform extraction method (Galla et al., 2019) at the University of Canterbury School of Biological Sciences. Extractions were assessed for quality by running 2 µl of DNA on a 2% agarose gel. A Qubit® 2.0 Fluorometer (Fisher Scientific) was used for DNA quantification.

Familial relationships are known for all samples collected, as they were sampled from birds of known provenance in captive conditions. However, to verify that no sample was mislabelled during genetic and genomic processing, parent–offspring relationships were verified through an allele mismatch exclusion analysis (Jones & Ardren, 2003) using microsatellite panels previously developed for kakī (Steeves et al., 2008) and kākāriki karaka (Andrews, 2013), with a maximum allowed mismatch of one allele at one locus (see *Microsatellite data* below). Family groups were further verified by clustering genome‐wide SNP relatedness values calculated using the KGD method (Dodds et al., 2015, see below) using principal component analysis and visualization of family groups using the TensorFlow Embedding Projector (Smilkov et al., 2016; *data not shown*).

### 2.2 Pedigree‐based relatedness

Multigenerational pedigrees were constructed for kakī and kākāriki karaka by entering studbook information (i.e., hatch date, sex, parentage and status) into the programme PopLink v. 2.5.1 (Faust, Bergström, Thompson, & Bier, 2018). Sex for all individuals was verified using molecular markers 2550F/2718R (Fridolfsson & Ellegren, 1999) for kakī and P2/P8 (Griffiths, Double, Orr, & Dawson, 1998) for kākāriki karaka, with PCR products run on a 3% agarose gel for visual characterization, with positive controls included. Due to the short distance between P2/P8 alleles on the Z and W chromosomes in kākāriki karaka (Robertson & Gemmell, 2006), 2 µl of PCR products using a tagged forward primer was combined with 11.7 µl formamide and 0.3 µl of GeneScan™ LIZ® 500 size standard (Applied Biosystems) and genotyped on an ABI 3739xl (Applied Biosystems), with alleles manually scored using GeneMarker v. 2.2 (State College, PA, USA). Inconsistencies in pedigrees were identified using the validation tool in PopLink and corrected using observations by DOC and the Isaac Conservation and Wildlife Trust. Pairwise estimates of kinship and inbreeding were produced using the programme PMx v. 1.6.20190628 (Lacy, Ballou, & Pollak, 2012), selecting only the individuals used in this study (*n* = 36 in kakī and kākāriki karaka) and treating all unknown individuals in the pedigree as wild founders. In order to produce direct comparisons of pairwise relatedness coefficients (*R*) between pedigree, genetic and genomic data, *R* was calculated from pedigree kinship data using *R*(*xy*)* =* 2 * *f*(*xy*)*/√*{(1 + *Fx*)(1 + *Fy*)}. In this formula, *f*(*xy*) is the kinship between two individuals (*x* and *y*) and *Fx* and *Fy* are the inbreeding coefficients of individuals *x* and *y* (Crow & Kimura, 1970).

### 2.3 Genetic‐ and genomic‐based relatedness

#### 2.3.1 Microsatellite data

Microsatellite loci (*n* = 8) previously described for kakī were amplified using PCR protocols by Steeves et al. (2008). Microsatellite loci (*n* = 17) designed for kākāriki karaka and one locus (*Cfor0809*) for Forbe's parakeet (*C. forbesi*) that cross‐amplified in kākāriki karaka were amplified using PCR protocols by Andrews et al. (2013) and Chan, Ballantyne, Lambert, and Chambers (2005), respectively. Samples were prepared for genotyping by adding 0.5 µl of PCR product to 11.7 µl formamide and 0.3 µl GeneScan™ LIZ® 500 size standard (Applied Biosystems) and were genotyped on either a 3130xl or 3730xl Genetic Analyser (Applied Biosystems). Chromatograms were visualized using GeneMarker v. 2.2. To avoid bias by potential dye shifts (Sutton, Robertson, & Jamieson, 2011), peaks were scored manually. The number of alleles and standard estimates of per locus diversity—including expected heterozygosity (*H*_O_) and observed heterozygosity (*H*_E_)—were produced using GenAlEx v. 6.5 (Peakall & Smouse, 2006; Smouse & Peakall, 2012). Tests for deviations from Hardy–Weinberg and linkage disequilibrium for these loci using samples that are representative of larger kakī and kākāriki karaka populations can be found in Steeves et al. (2008), Steeves, Maloney, Hale, Tylianakis, and Gemmell (2010) and Andrews (2013), respectively. For kākāriki karaka, only eight of the 18 microsatellite markers previously described were polymorphic in this study and these eight loci were used in all downstream analyses.

Genetic‐based *R* estimates were produced in the programme COANCESTRY v. 1.0.1.9 (Wang, 2011). COANCESTRY offers seven different estimators of relatedness, and to choose the most appropriate estimator for the kakī and kākāriki karaka microsatellite data sets, we employed the simulation module within COANCESTRY using allele frequencies, missing data and error rates from our microsatellite data sets. To produce dyads that represent the relationships and degree of inbreeding found within kakī and kākāriki karaka, we used R package “identity” (Li, 2010) to generate 10,879 dyads for kakī and 1,484 dyads for kākāriki karaka based on the pedigrees of both species. The frequency of each unique dyad in the kakī and kākāriki karaka data sets was scaled to create 1,000 dyads for each set that are representative of relationships between individuals used in this study. The COANCESTRY simulations were conducted using allele frequencies, error rates and missing data rates from each microsatellite data set, with settings changed to account for inbreeding. The triadic likelihood approach (Wang, 2007) was selected given it had the highest Pearson's correlation with “true” relatedness for both data sets (see Supporting Information for details). This approach is also preferred, as it is one of the few estimators that accounts for instances of inbreeding (Wang, 2007).

To estimate *R* with our genetic data set, COANCESTRY programme parameters were set to account for inbreeding, with the number of reference individuals and bootstrapping samples set to 100.

#### 2.3.2 Genomic data

##### Reference genomes

A reference genome for kakī has already been assembled (Galla et al., 2019) and was used in this study. To assemble a de novo reference genome for kākāriki karaka, a paired‐end library was prepared at the Institute of Clinical Molecular Biology (IKMB) at Kiel University using the Nextera™ DNA Flex Library Prep Kit according to the manufacturer's specifications and sequenced on an Illumina NovaSeq™ 6,000 with 2 × 150 bp reads at a depth of approximately 70×.

FastQC v. 0.11.8 (Andrews, 2010) was used to evaluate the quality of the raw Illumina data and assess potential sample contamination. Initial read trimming was performed using TrimGalore v. 0.6.2 (Krueger, 2019) and Cutadapt v. 2.1 (Martin, 2011) with an end trim quality of 30, a minimum length of 54 and using the *‐‐nextseq* two‐colour chemistry option. A median Phred score of 20 was also used for initial read trimming to remove obvious data errors; it should be noted that the assembly programmes used here (i.e., Meraculous‐2D v. 2.2.10 and MaSuRCA v. 3.2.9; see below) have their own error corrections embedded in their respective pipelines. Kmer analyses were performed using Jellyfish v. 2.2.10 (Marçais & Kingsford, 2011) prior to assembly to assess heterozygosity and contamination. Two genome assembly programmes were tested for assembly performance: Meraculous‐2D v. 2.2.5.1 (Chapman et al., 2011) and MaSuRCA v. 3.2.9 (Zimin et al., 2013). Meraculous was run using trimmed reads in diploid mode 1, with all other assembly parameters set to default. MaSuRCA was run using untrimmed reads, as it incorporates its own error correction pipeline. MaSuRCA parameter adjustments include a grid batch size of 300,000,000, the longest read coverage of 30, a Jellyfish hash size of 14,000,000,000 and the inclusion of scaffold gap closing; all other parameters were set to default for nonbacterial Illumina assemblies. The final assembly using the Meraculous pipeline was more fragmented (i.e., an N50 of 28.5 kb with 67,046 scaffolds > 1 kb), while the MaSuRCA genome was less fragmented (i.e., an N50 of 107.4 kb with 66,212 scaffolds > 1 kb) but contained possible artefacts due to heterozygosity (i.e., tandem repeats flanking short stretches of “N”s). To correct for these issues, the Meraculous assembly was first aligned to the MaSuRCA assembly using Last v. 959 (Kielbasa, Wan, Sato, Horton, & Frith, 2011); then, alignments were filtered to find matches where the Meraculous assembly spans the entirety of the tandem repeat in the MaSuRCA scaffolds, but lacking the tandem repeat or stretch of “N”s (i.e., gaps). In those cases, the aligned sequence in the MaSuRCA scaffold was replaced with the Meraculous match. All compute requirements needed to assemble the kākāriki karaka genome are available in Supporting Information.

##### Whole‐genome resequencing

Kakī resequencing libraries were prepared at IKMB using a TruSeqⓇ Nano DNA Library Prep Kit following the manufacturer's protocol and were sequenced across 34 lanes of an Illumina HiSeq 4,000. Twenty‐four individuals were sequenced at high coverage depth (approximately 50×) for an aligned study, and all others were sequenced at a lower coverage depth (approximately 10×). Kākāriki karaka libraries were prepared at IKMB using the Nextera™ DNA Flex Library Prep Kit according to the manufacturer's specifications and sequenced across one lane of an Illumina NovaSeq™ 6,000 at IKMB at a coverage depth of approximately 10×, with one individual sequenced at a depth of approximately 70×, which was additionally used for the reference genome (see above).

FastQC v. 0.11.4 and 0.11.8 (Andrews, 2010) were used to evaluate the quality of the raw Illumina data for kakī and kākāriki karaka, respectively. Kakī resequencing reads were subsequently trimmed for the Illumina barcode, a minimum Phred quality score of 20 and a minimum length of 50 bp using Trimmomatic v. 0.38 (Bolger, Lohse, & Usadel, 2014). Because kākāriki karaka libraries were produced using different library preparation protocols and nextera chemistry, reads were trimmed using TrimGalore v. 0.6.2 (Krueger, 2019) for nextera barcodes and two‐colour chemistry, using a median Phred score of 20, end trim quality of 30 and a minimum length of 54. Prior to mapping, the kakī reference genome was concatenated to a single chromosome using the custom perl script “concatenate_genome.pl” (Moraga, 2018a) for use in an aligned project that used both resequencing and genotyping‐by‐sequencing reads (see Galla et al., 2019). The kakī and kākāriki karaka reference genomes were indexed, and resequencing reads were mapped using Bowtie2 v. 2.2.6 and v. 2.3.4.1 (Langmead & Salzberg, 2012), respectively, with the setting *‐‐very‐sensitive*. Resulting SAM files were converted to BAM and were sorted using Samtools v. 1.9 (Li et al., 2009). Read coverage and variant calling were performed using *mpileup* in BCFtools v. 1.9 (Li et al., 2009). The custom perl script “split_bamfile_tasks.pl” (Moraga, 2018b) was used to reduce the computational time needed for mpileup by increasing parallelization. SNPs were detected, filtered and reported using BCFtools. Filtering settings were set to retain biallelic SNPs with a minor allele frequency (MAF) greater than 0.05, a quality score greater than 20 and a maximum of 10% missing data per site. After a series of filtering trials for each species (see Supporting Information for details), depth for kakī was set to have an average mean depth greater than 10, while kākāriki karaka depth was set so that each site had a minimum depth of 5 and a maximum depth of 200. Resulting SNPs for both data sets were pruned for linkage disequilibrium using BCFtools with the *r^2^* set to 0.6 and a window size of 1,000 sites. Sites were not filtered for Hardy–Weinberg equilibrium, as the nature of these data sets (mostly family groups) violates the assumptions of random mating. Per site missingness, depth and diversity—including proportion of observed and expected heterozygous SNP sites per individual, nucleotide diversity and SNP density per kb—were evaluated in the final sets using VCFtools v. 1.9 (Danecek et al., 2011). Diversity statistics were calculated using polymorphic markers only.

##### SNP‐based relatedness

To produce estimates of *R* using whole‐genome SNPs, the programme KGD (Dodds et al., 2015) was used, as it was designed to estimate relatedness using reduced representation and resequencing data while taking into account read depth. Pairwise *R* values derived from KGD were scaled so that self‐relatedness for all individuals was equal to 1 using the formula *MS = D* × *MO* × *D* where *MS* is the scaled matrix, *MO* is the original matrix, and *D* is a diagonal matrix with elements *D = 1/√(diag(MO))*. This scaling was performed to simplify downstream Mantel tests by creating a standardized diagonal value. This scaling was maintained throughout all analyses, as the scaled approach better approximated parent–offspring relationships, while demonstrating minimal bias to downstream analyses (see Supporting Information for details).

We evaluated the scaled KGD approach with other maker‐based relatedness estimators, including the triadic likelihood approach (Wang, 2007), the KING estimator (Waples, Albrechtsen, & Moltke, 2019) and the *r_xy_* method (Hedrick & Lacy, 2015), using parent–offspring relatedness as a benchmark for precision. We found that the scaled KGD approach estimates parent–offspring relatedness closer to 0.5 compared to other relatedness estimators, while still providing estimates that are significantly concordant with all other approaches in both kakī (Pearson's *r* = 0.80–0.96, *p* < .001) and kākāriki karaka (Pearson's *r* = 0.89–96, *p* < .001; see Supporting Information for details).

### 2.4 Comparison of relatedness

Mantel tests using the R package *ape* (Paridis & Schliep, 2018) were performed with 1,000 iterations to determine whether pedigree‐, microsatellite‐ and SNP‐based *R* were significantly correlated compared to a null distribution. Pearson's correlation coefficient (*r*) was additionally calculated to provide an additional measure of concordance between approaches. While our relatedness data sets are nonparametric, Pearson's was used over nonparametric tests, such as rank correlations, as our pedigree and microsatellite data sets have an excess of tied values.

### 2.5 Pairing recommendations

We used two complementary methods in PMx v. 1.6.20190628 (Lacy et al., 2012) to determine whether pairing recommendations change using pedigree‐, microsatellite‐ and SNP‐based approaches for estimating *R*. First, we used mate suitability index (MSI), which scores how valuable offspring of a potential pair would be by taking into account four factors: deltaGD (i.e., the net positive or negative genetic diversity provided to the population), the difference of mean kinship values of the pair, the inbreeding coefficients of resulting offspring and the extent of unknown ancestry (Ballou, Earnhardt, & Thompson, 2001; Lacy et al. 2012). MSI scores scale from 1 to 6, with 1 being “very beneficial,” and 6 being “very detrimental.” An additional category denoted with a “‐” indicates “very highly detrimental” pairings. Here, we assign this category with a numerical MSI score of 7. MSI scores provide a standardized approach for comparing pairing recommendations within and among species, including those based on the three approaches used in this study. However, Ballou et al. (2001) recommend caution when using automated pairing recommendations such as MSI in small and inbred populations. Thus, we also used mean kinship (MK) rank, which is an approach known to perform well in small and inbred populations (Ballou & Lacy, 1995; Rudnick & Lacy, 2008). This approach ranks individuals from lowest to highest MK among males and females, thereby creating a list of individuals for pairing prioritization.

For MSI score and MK rank analyses, only the individuals used in this study (*n* = 36 for both kakī and kākāriki karaka) were selected for analysis. PMx settings were set to default, with the exception of treating all unknown individuals in the pedigree as wild (i.e., 100% analytics known in the pedigree) to minimize bias from unknown pedigree assignments. Pedigree‐based MSI scores and MK ranks were produced using pedigree‐based kinship, while pairing recommendations using microsatellites and SNPs were produced using coefficients of relatedness. These genetic and genomic estimates of relatedness were uploaded to PMx, which divides these values by two to create an empirical metric of kinship. These empirical values were weighted to 1 to produce MSI scores and MK ranks that relied only on empirical data.

Pearson's correlation (*r*) was used to evaluate whether pairwise MSI scores and MK ranks between approaches were concordant. To test whether the distribution of MSI scores was statistically different from one another, we used a nonparametric Kruskal–Wallis test with Bonferroni correction and a Tukey honest significant difference test.

## 3 RESULTS

### 3.1 Pedigree‐based relatedness

**Figure 2:**
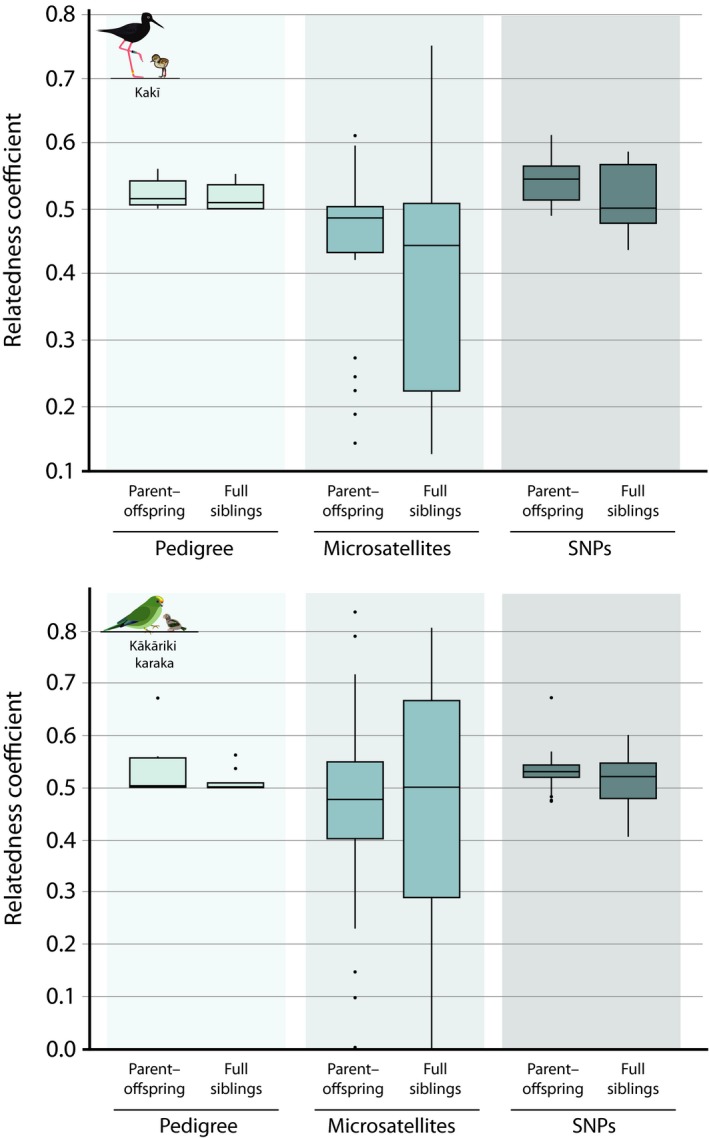
Parent–offspring and full sibling relatedness values derived from pedigree‐ (pale blue), microsatellite‐ (medium blue) and SNP‐based (dark blue) methods in kakī (top graph) and kākāriki karaka (bottom graph) for the 36 focal individuals in this study

This study has produced the first multigenerational pedigrees for two critically endangered endemic birds from Aotearoa. The kakī pedigree includes 2,481 wild and captive individuals recorded from 1977 to present, with a pedigree depth ranging from 1 to 8 generations (3.35 average). The number of founders and founder genome equivalents in the kakī pedigree (94 and 12.9, respectively) is high relative to the kākāriki karaka pedigree (16 and 12, respectively), and the % known ancestry is lower (55% and 100%, respectively; Table 2). Pedigree‐based *R* between the 36 focal kakī ranged from 0 to 0.56, with an average *R* of 0.13 ± *SD* 0.13. The average coefficient of relatedness between all known kakī parent–offspring was higher than the expected 0.5 contribution from each parent (0.52 ± *SD* 0.02), with averaged full sibling *R* of 0.52 ± *SD* 0.02 (Figure 2). The kākāriki karaka pedigree includes 624 captive individuals from 2003 to present, with an a pedigree depth ranging from 1 to 5 generations (2.48 average). Pedigree‐based *R* for the 36 focal kākāriki karaka ranged from 0 to 0.67, with an average *R* of 0.19 ± *SD* 0.18. Average *R* between all parent–offspring was 0.52 ± *SD* 0.03, with averaged full sibling *R* being 0.51 ± *SD* 0.02 (Figure 2).

**Table 2:** Descriptive statistics based on pedigree data, as produced by PMx, including number of individuals, sex ratio (% males), maximum age, gene diversity, number of founders, number of founder genome equivalents, average inbreeding, average mean kinship, average generation time, % ancestry and analytic known, and effective population size

Pedigree statistic	All pedigreed individuals		Individuals in study	
	Kakī	Kākāriki Karaka	Kakī	Kākāriki Karaka
No. of Individuals	2,481	618	36	36
Sex ratio	0.27^a^	0.5	0.44	0.5
Max. age (years)	24	16.4	19.3	19.6
Gene diversity	0.96	0.915	0.9112	0.886
No. of founders	94	16	29	12
Founder genome equivalents	12.9	12	5.6	4.4
Average inbreeding	0.027	0.03	0.034	0.016
Average mean Kinship	0.039	0.085	0.089	0.114
Average generation time	4.82	1.31	5.25	3.79
% Ancestry known	55	100	58	100
% Analytic known	100	100	100	100
Ne/N	0.103	0.072	0.353	0.541

a The sex ratio for all pedigreed individuals for kakī is biased by a large number of individuals with unknown sex.

### 3.2 Microsatellite diversity and relatedness

All eight microsatellite markers for kakī successfully amplified in all individuals used in this study. The number of alleles present across kakī loci ranged from 2 to 4 (average 3.13 ± *SD* 0.64; Table 3), with overall fewer alleles found here than reported in previous studies utilizing these loci with more individuals (Hagen, Hale, Maloney, & Steeves, 2011; Steeves et al., 2010). While 18 microsatellite markers were amplified in kākāriki karaka, one was removed from this study for not successfully amplifying in more than 50% of individuals (locus *OFK56*) and nine were removed for being monomorphic (Table 3). The number of alleles among polymorphic loci ranged from 2 to 4 (average 3.0 ± *SD* 0.93), with overall fewer alleles found here than reported in previous studies (Andrews, 2013; Andrews et al., 2013). Observed (*H*_O_) heterozygosity and expected (*H*_E_) heterozygosity for kakī (average *H*_O_ = 0.57 ± *SD* 0.17, average *H*_E_ = 0.54 ± *SD* 0.14) were higher than kākāriki karaka (average *H*_O_ = 0.43 ± *SD* 0.23, average *H*_E_ = 0.43 ± *SD* 0.20; Table 3).

**Table 3:** Descriptive statistics, including number of alleles, observed heterozygosity (*H*_O_) and expected heterozygosity (*H*_E_) for microsatellite loci used in this study. Loci from kākāriki karaka that were monomorphic (OFK12, OFK 19, OFK21, OFK26, OFK31, OFK33, OFK52, OFK56, OFK58, OFK61) are not included

Species	Locus	No. of alleles	*H_O_*	*H_E_*
Kakī	BS2	3	0.667	0.652
	BS9	3	0.611	0.59
	BS12	3	0.278	0.245
	BS13	2	0.528	0.5
	BS21	4	0.833	0.703
	BS27	4	0.667	0.551
	BS40	3	0.444	0.448
	BSdi7	3	0.556	0.596
Kākāriki Karaka	OFK9	4	0.472	0.477
	OFK41	4	0.75	0.702
	OFK50	3	0.444	0.513
	OFK54	4	0.722	0.574
	OFK55	2	0.278	0.346
	OFK60	2	0.222	0.239
	OFK62	2	0.083	0.08
	C for 809	3	0.44	0.52

Microsatellite‐based *R* between all kakī used in this study ranged from 0 to 0.85, with an average *R* of 0.16 ± *SD* 0.19. Average *R* between all known kakī parent–offspring (0.44 ± *SD* 0.13) was below the minimum expected relatedness value of 0.5, with a larger standard deviation of *R* values compared to pedigree‐based estimates. Averaged microsatellite‐based full sibling *R* (0.41 ± *SD* 0.20) also had a larger deviation around the mean compared to the parent–offspring estimates (Figure 2).

Microsatellite‐based *R* between all kākāriki karaka used in this study ranged from 0 to 0.84, with an average *R* of 0.18 ± *SD* 0.22. Similar to kakī, average *R* between all known kākāriki karaka parent–offspring relationships (0.47 ± *SD* 0.19) was below the minimum expected *R* value of 0.5, with a larger standard deviation of *R* values compared to pedigree‐based estimates. Averaged full sibling *R* (0.49 ± *SD* 0.21) also had a larger deviation around the mean compared to microsatellite‐based parent–offspring estimates (Figure 2).

### 3.3 Reference genome assembly, SNP discovery, diversity and relatedness estimates

#### 3.3.1 Kākāriki karaka reference genome assembly

Reference genome library preparation and Illumina NovaSeq™ sequencing resulted in 584.47 million total reads for the kākāriki karaka genome. The final kākāriki karaka genome assembly was 1.15 GB in length, which is within the range of most assembled avian genomes (e.g., Zhang et al., 2014). The final assembly had 66,212 scaffolds with a scaffold N50 of 107.4 kb. See Data Availability section for access information.

#### 3.3.2 SNP discovery and diversity

Library preparation and Illumina sequencing resulted in 6.07 billion total reads for kakī (168.69 ± *SD* 65.32 million reads). In addition to the individual used for the reference assembly, 3.64 billion total reads (average = 103.92 ± *SD* 29.76 million reads) were produced for the additional 35 kākāriki karaka in this study. More SNPs were discovered during initial SNP discovery using kākāriki karaka than kakī, and more remained postfiltering (Table 4). These filtered SNPs were used for all downstream analyses. Average missingness was low for both data sets (Table 4), but lower for kākāriki karaka than kakī, as kākāriki karaka had a hard minimum cut‐off for depth during filtering that resulted in no missing data. Average depth for both data sets was relatively high (Table 4), with kakī having slightly higher average depth. Average diversity statistics (nucleotide diversity, and the average observed and expected SNP heterozygosity per individual postfiltering) were similar in both species, with diversity in kakī being slightly higher. SNP density using the kakī data set was higher than the kākāriki karaka data set (Table 4).

**Table 4:** Descriptive statistics, including number of SNPs pre‐ and postfiltering, average depth ± *SD*, average missingness ± *SD*, average nucleotide diversity (*π*) ± *SD*, average proportion of observed heterozygous SNP sites (*H*_O_) ± *SD*, average proportion of expected heterozygous SNP sites (*H*_E_) ± *SD* and average SNP density (number of SNPs per kilobase) ± *SD*

Species	No. of SNPs prefiltering	No. of SNPs postfiltering	Average depth	Average missingness	Average *π*	Average *H_O_* of SNP sites	Average *H_E_* of SNP sites	SNP density
Kakī	4,246,100	68,144	28.73 ± 10.29	0.002 ± 0.004	0.35 ± 0.14	0.40 ± 0.02	0.35 ± 0.00	0.58 ± 3.18
Kākāriki Karaka	22,435,128	90,949	25.1 ± 14.87	0.00 ± 0.00	0.33 ± 0.14	0.37 ± 0.02	0.33 ± 0.00	0.17 ± 0.42

#### 3.3.3 SNP‐based relatedness

SNP‐based *R* between all kakī used in this study ranged from 0.13 to 0.61, with an average *R* of 0.27 ± *SD* 0.09. Similar to pedigree‐based estimates, average *R* between all known kakī parent–offspring was slightly higher than the expected relatedness value of 0.5 with a small standard deviation relative to microsatellite‐based estimates (0.54 ± *SD* 0.03). Averaged full sibling *R* also had a larger deviation around the mean (0.52 ± *SD* 0.05) than parent–offspring relationships (Figure 2).

SNP‐based *R* between all kākāriki karaka used in this study ranged from 0.08 to 0.67, with an average *R* of 0.30 ± *SD* 0.12. Similar to pedigree‐based estimates, average *R* between all known kākāriki karaka parent–offspring was slightly above the expected *R* value of 0.5 with a small standard deviation relative to genetic‐based estimates (0.53 ± *SD* 0.03). Averaged full sibling relatedness also had a larger deviation around the mean (0.52 ± *SD* 0.05) compared to the pedigree‐based estimates (Figure 2).

### 3.4 Comparison of relatedness estimates and pairing recommendations

**Figure 3:**
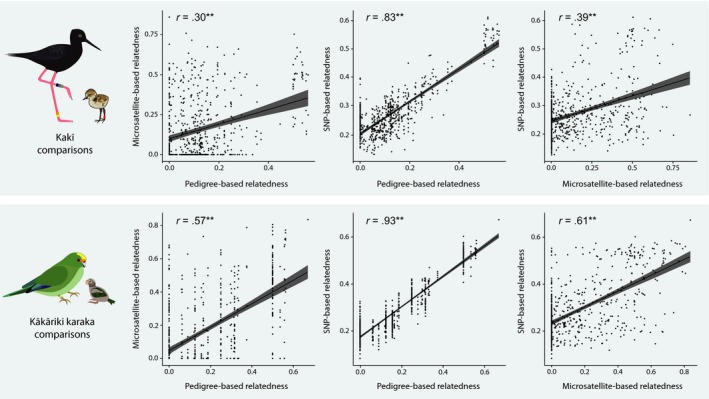
Scatterplots showing relationships between pedigree‐, microsatellite‐ and SNP‐based relatedness estimates in known family groups for kakī and kākāriki karaka. A trend line (black) and 95% confidence intervals (grey) are shown in each comparison

All kakī and kākāriki karaka *R* estimates using pedigree‐, microsatellite‐ and SNP‐based approaches correlated with one another with high statistical significance (Mantel test, *p* < .001; Pearson's correlation, *p* < .001; Figure 3). Of all the approaches, the correlation coefficient between pedigree‐ and SNP‐based approaches was markedly higher than between other approaches, indicating that they are the most concordant (Figure 3).

**Figure 4:**
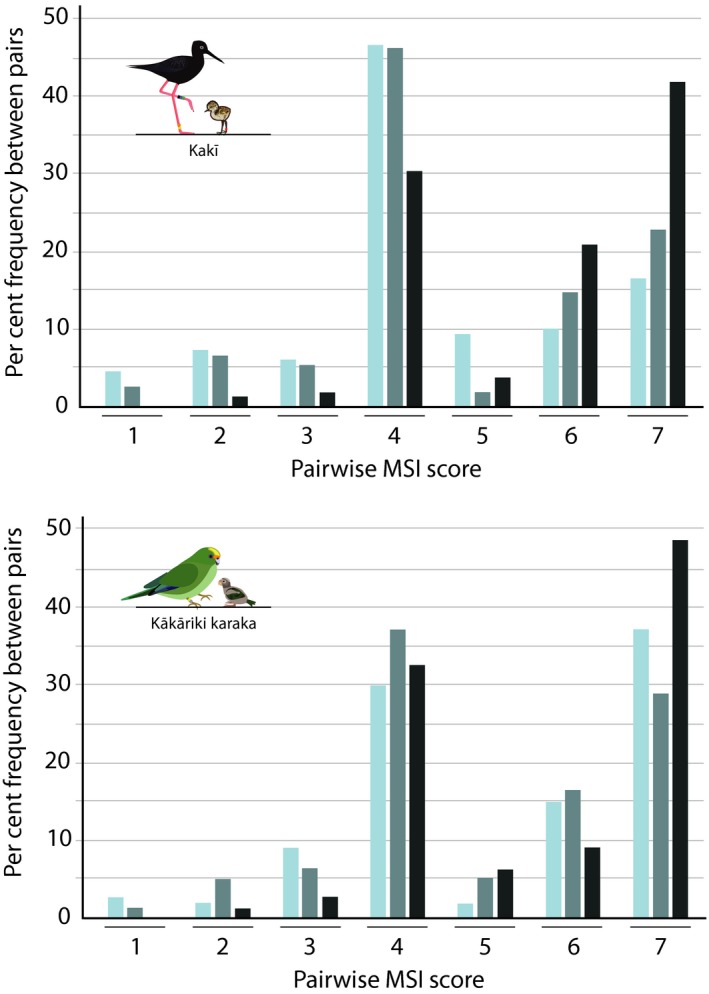
Frequency of MSI scores using pedigree‐ (pale blue), microsatellite‐ (medium blue) and SNP‐based (dark blue) kinship/relatedness values in kakī and kākāriki karaka. MSI, mate suitability index; SNP, single nucleotide polymorphism

Mate suitability index scores and MK ranks were calculated as an approximation for pairing recommendations derived from *R* estimates using the different approaches. Average pedigree‐based MSI scores for kakī (4.46 ± *SD* 1.59) were lower on average than microsatellite‐based scores (4.73 ± *SD* 1.63), but not significantly different from each other (Kruskal–Wallis test with Bonferroni correction, *p* = .2). SNP‐based MSI scores for kakī (average = 5.67 ± *SD* 1.39) were significantly higher than pedigree‐ and microsatellite‐based scores (Kruskal–Wallis test with Bonferroni correction, *p* < .001), with SNP‐based scores providing the highest frequency of category 7 (i.e., very highly detrimental) pairings (Figure 4). While the distributions of MSI scores between each approach were different, each approach produced scores that correlated significantly with one another (Pearson's correlation, *p* < .01–0.001). Similar to correlations between *R* estimates, correlation coefficients between pedigree‐ and SNP‐based MSI scores were highest (Pearson's *r* = 0.5, see Figure S1 for details). Of the 320 possible kakī pairings, 38% did not experience a change in MSI score value between pedigree‐ and SNP‐based approaches; however, 20% of pairings experienced an MSI score change that was 3+ categories different. In 2% of pairings, pedigree‐based MSI scores were categorized as a 1 (i.e., preferred pairing), while SNP‐based MSI scores were categorized as a 7 (i.e., very highly detrimental). Correlations between MK ranks provided by the three approaches were significant between pedigree‐ and SNP‐based approaches only (Pearson's *r* = 0.75, *p* < .001; see Figure S2 for details). Among pedigree‐ and SNP‐based MK ranks, 64% of individuals experienced a minimal rank shift of 0–3 categories, 22% experienced a moderate rank shift of 4–7 categories, and 3% experienced a high rank shift of ≥8 categories.

Similar to kakī, average kākāriki karaka SNP‐based MSI scores (5.64 ± *SD* 1.47) were significantly higher than pedigree (5.20 ± *SD* 1.71) and microsatellite (5.04 ± *SD* 1.61) scores (Kruskal–Wallis test with Bonferroni correction, *p* < .001), while pedigree‐ and microsatellite‐based scores did not significantly differ (Kruskal–Wallis test with Bonferroni correction, *p* = .67). SNP‐based scores provided the highest frequency of category 7 (i.e., very highly detrimental) pairings (Figure 4). Each approach also produced scores that correlated significantly with one another (Pearson's correlation, *p* < .001), with the highest correlation coefficients seen between pedigree‐ and SNP‐based MSI scores (Pearson's *r* = 0.65, see Figure S1 for details). Of the 324 possible pairings for kākāriki karaka, 59% did not experience a change in MSI score value between pedigree‐ and SNP‐based approaches; however, 9% of pairings experienced an MSI score change that was 3+ categories different. In 2% of pairings, pedigree‐based MSI scores were categorized as a 1 (i.e., very beneficial), while SNP‐based MSI scores were categorized as a 7 (i.e., very highly detrimental). Correlations between MK rank were significant between pedigree‐ and SNP‐based approaches (Pearson's *r* = 0.64, *p* < .001) and microsatellite‐ and SNP‐based approaches (Pearson's *r* = 0.51, *p* = .002, see Figure S2 for details). Among pedigree‐ and SNP‐based MK ranks, 53% of individuals experienced a minimal rank shift of 0–3 categories, 31% experienced a moderate rank shift of 4–7 categories, and 8% experienced a high rank shift of ≤8 categories.

## 4 DISCUSSION

This study is the first to compare pedigree‐, microsatellite‐ and SNP‐based estimates of relatedness and subsequent pairing recommendations for conservation breeding programmes. The results indicate that microsatellites provide the least precision when estimating relatedness in known family groups, with pedigree‐ and SNP‐based estimates providing higher precision and a much closer approximation of parent–offspring and full sibling relatedness. Further, estimates of relatedness and downstream pairing recommendations using MSI scores and MK ranks are both more concordant when using pedigree‐ and SNP‐based data sets compared to microsatellite‐based data sets. Despite this, there were important differences in pairing recommendations between pedigree‐ and SNP‐based approaches, with SNP‐based MSI scores being statistically higher than pedigree‐based scores, and some substantial disagreements existing between the two sets of MSI scores and MK ranks. Together, this study provides insight into the differences between pedigree‐, microsatellite‐ and SNP‐based approaches for making pairing recommendations and a pathway for estimating relatedness using genome‐wide SNPs to inform pairing decisions in poorly pedigreed conservation breeding programmes worldwide.

### 4.1 Relatedness comparisons

When producing empirical estimates of relatedness, simulations were performed to choose the most suitable estimator for microsatellites, and various relatedness estimators and filtering schemes for SNPs were evaluated to find the approach that best approximated known parent–offspring relationships as a biologically relevant benchmark. While different relatedness estimators and filtering schemes will result in different point estimates of relatedness, this study demonstrates an approach for producing relatedness estimates that are well suited for our particular data set and research question.

Pedigree‐based estimates of parent–offspring and full sibling relatedness approximated 0.5 for both kakī and kākāriki karaka (Figure 2), with some measures being slightly higher, which likely reflects intergenerational inbreeding and/or higher pedigree depth from the baseline (reference) population. These results are consistent with expectations, as pedigrees are based on the probability of Mendelian inheritance, which postulates that first‐order relationships (i.e., parents and offspring, and siblings) share 50% of their genomic information (Lacy, 1995; Wright, 1922). We expect realized (i.e., empirical) parent–offspring relationships to also approximate 0.5, but a broader range of realized relatedness estimates among full siblings, as they may receive different genetic material from each parent due to recombination and independent assortment during meiosis and random fertilization (Hill & Weir, 2011, 2012; Speed & Balding, 2015). Even when pedigrees are robust, this study highlights an unavoidable shortcoming as pedigrees do not adequately capture true relatedness between full siblings. We anticipate this uncaptured diversity may prove useful for maximizing existing diversity, especially in conservation breeding programmes with relatively few founders (Ballou & Lacy, 1995).

Compared to the pedigree‐based approach, our empirical data sets (i.e., microsatellites and SNPs) capture more variation between siblings than parents and offspring (Figure 2). A broad range of microsatellite‐based relatedness estimates were observed in both parent–offspring and sibling relationships, compared to the SNP‐based approach. In some instances, even parent–offspring pairings appeared relatively unrelated using microsatellites (e.g., minimum parent–offspring *R* = 0.14 in kakī and *R* = 0 in kākāriki karaka), which underscores the lack of precision in this approach and how it could inadvertently lead to poorly informed pairing recommendations. These large ranges of relatedness values using microsatellites can be explained because genetic‐based relatedness values between parent–offspring and full siblings are based on allele frequencies, and relatedness between individuals that share common alleles will be lower than individuals that share rare alleles (Speed & Balding, 2015; Wang, 2011). This bias in relatedness values can be exacerbated when samples sizes are small (Wang, 2017), which is typical of conservation breeding programmes. Furthermore, the lack of precision using microsatellites shown here is consistent with studies that suggest relatively few markers with low allelic diversity are insufficient for estimating relatedness and inbreeding, especially in genetically depauperate species (e.g., Attard et al., 2018; Escoda et al., 2017; Hellmann et al., 2016; Taylor, 2015; Taylor et al., 2015).

Compared to microsatellite‐based relatedness, SNP‐based relatedness showed a relatively small range with parent–offspring and full sibling relatedness estimates approximating 0.5, and full siblings showing a wider range of values than parent–offspring relationships (Figure 2). Not only is this pattern consistent with expectations given the behaviour of chromosomes during meiosis and random fertilization, but it also shows more precision than the microsatellite data sets. Other researchers have found similar results in a diverse range of wild taxa, indicating that thousands of genome‐wide SNPs show more precision than microsatellites when measuring relatedness and inbreeding (e.g., Attard et al., 2018; Hellmann et al., 2016; Hoffman et al., 2014; Lemopoulos et al., 2019; Thrasher, Butcher, Campagna, Webster, & Lovette, 2018).

Beyond parent–offspring and full sibling relationships, pedigree‐ and SNP‐based relatedness estimates showed the highest concordance with one another among the three approaches used (Figure 3). In kakī, the data sets used here include non‐captive‐bred individuals with intensively monitored wild parents. These results provide more credibility to the semi‐wild kakī pedigree, where socially monogamous wild pairs of kakī are assumed to be the genetic parents of offspring at nests (but see also Overbeek et al., 2017). Still, it should be noted that many pairs with pedigree‐based relatedness values of 0 had SNP‐based relatedness values ranging upwards of 0.40 in kakī 0.33 and in kākāriki karaka, which approximates first‐ and second‐order relationships in both species (Figure 2). This indicates that pedigree‐based *R* between these individuals may be downwardly biased by the assumption that no variance in relatedness exists among founders, missing information and/or low pedigree depth (Balloux et al., 2004; Bérénos et al., 2014; Hammerly et al., 2016; Hogg et al., 2019; Kardos et al., 2015; Lacy, 1995; Pemberton, 2008; Rudnick & Lacy, 2008; Tzika et al., 2009).

### 4.2 Pairing recommendations

When these relatedness values are translated into pairing recommendations using MSI scores and MK rank, there is a high concordance between pedigree‐ and SNP‐based approaches, with SNP‐based MSI scores being significantly higher than pedigree‐ and microsatellite‐based approaches. The latter result is somewhat expected, given that average relatedness estimates using SNPs was highest among the approaches used here, and empirical estimates of relatedness and inbreeding are usually higher than pedigrees as they more effectively capture relatedness between founders or misassigned individuals (Hammerly et al., 2016; Hogg et al., 2019). With that said, when making pairing recommendations using kinship‐based pairing decisions (e.g., Ballou & Lacy, 1995), it is often the relative kinships between individuals that are more important than absolute values (Galla et al., 2019; McLennan, Wright, Belov, Hogg, & Grueber, 2019). This suggests that pedigree‐ and SNP‐based approaches both yield similar results for pairing recommendations, with some important differences. For example, while correlation coefficients between these two sets of MSI scores are high relative to other comparisons, there are instances where pairings are considered “highly beneficial” (i.e., MSI category 1) when using the pedigree and “very highly detrimental” (i.e., MSI category 7) when using SNPs. When comparing MK ranks between pedigree‐ and SNP‐based approaches, some kakī and kākāriki karaka experienced large shifts in rank (i.e., ≥8 positions difference) depending on the approach used. Although we expect some differences between pedigree‐ and SNP‐based MSI scores and MK ranks, we attribute these very large differences to errors in the pedigree (e.g., Hammerly et al., 2016) or violations of the assumption that there is no variance in founder relationships (e.g., Hogg et al., 2019). Of all kakī and kākāriki karaka pairings that experienced a large shift between pedigree‐ and SNP‐based MSI scores, most feature recurring individuals with wild parentage (i.e., founders), and in one recurring occasion, a wild individual (kakī) with high pedigree depth that likely represents an entry error in the pedigree.

### 4.3 Management implications

Pedigree‐, genetic‐ and genomic‐based tools each have their advantages to inform conservation management. For example, pedigrees capture both genetic and demographic considerations dating back to the founding of the population, while empirical estimates of relatedness can circumvent pedigree errors and issues surrounding founder relationships by expressing realized relatedness between all sampled individuals. From the results shown here, we recommend that when conservation breeding programmes are poorly pedigreed (i.e., pedigrees of low depth or containing missing data), SNPs should be incorporated to provide a precise indicator of relatedness to genetically inform pairing decisions. The microsatellite panels used here have shown low precision in estimating relatedness, with demonstrated downstream effects for pairing recommendations compared to pedigree‐ and SNP‐based approaches. More microsatellites could be developed to mitigate this shortcoming; however, other studies indicate that a larger number microsatellites (e.g., 20–40 markers) may not equate to higher precision for relatedness estimates and inbreeding coefficients, especially in threatened species with low allelic diversity (Nietlisbach et al., 2017; Robinson et al., 2013; Taylor, 2015; Taylor et al., 2015). Further, the time and cost associated with building larger microsatellite panels and generating microsatellite data will likely be surpassed by the production of genome‐wide SNPs, either by a whole‐genome resequencing approach as shown in this study or by a reduced representation sequencing approaches (e.g., RAD sequencing, or genotyping‐by‐sequencing; Galla et al., 2016; Narum, Buerkle, Davey, Miller, & Hohenlohe, 2013). Currently, for kakī and kākāriki karaka, reduced representation sequencing is more cost‐effective than whole‐genome resequencing (i.e., approximately one‐third of the price, depending on the genome size, as of 2019)—but we foresee more people shifting towards whole‐genome resequencing in the near future, given the decreasing cost of high‐throughput sequencing (Hayden, 2014) and the ability to ask more research questions using whole‐genome resequencing data sets (see Future Directions below for details). This is particularly true for birds, whose genomes are small (e.g., 1.05–1.26G) relative to many vertebrates (Zhang et al., 2014).

We anticipate SNPs will be particularly applicable in circumstances when pedigrees are the least reliable. For instance, when the founders of a conservation breeding population have no ancestry data available and are likely to be related, SNP‐based relatedness estimates between individuals can be used to avoid highly related matings (Hogg et al., 2019). This situation may not only coincide with the original founding event of a captive population, but iteratively when individuals are sourced from wild or translocated populations to augment the captive population, as suggested in Frankham (2008) and Hogg et al. (2019). For example, in kākāriki karaka, whole‐genome resequencing has been made available for all current breeding individuals in the conservation breeding programme, including individuals who are founders themselves. Because birds of unknown ancestry are being routinely sourced from highly endangered wild populations, and will also be founders, we anticipate the need for resequencing these birds as they are incorporated into the breeding programme to assess their relatedness to other individuals. In addition to traditional captive‐bred populations (i.e., ex situ management), this approach is applicable to intensively managed wild populations (i.e., in situ management), where robust pedigrees are lacking, but conservation translocations can be informed by relatedness between individuals in a managed landscape (e.g., kākāpō, *Strigops habroptilus*, Elliott, Merton, & Jansen, 2001; scimitar‐horned oryx, *Oryx dammah*, Wildt et al., 2019).

While we expect SNPs will be important for pairing recommendations moving forward, we do not expect they will eclipse well‐established pedigrees, as both approaches have advantages for conservation breeding. Instead, we envision a combined approach where realized relatedness from SNPs can be used to augment data‐rich pedigrees. With that said, there are relatively few studies to date that effectively combine existing pedigree data with genomic estimates of relatedness to inform pairing recommendations (but see Hogg et al., 2019; Ivy, Putnam, Navarro, Gurr, & Ryder, 2016). To date, these studies are largely limited to SNPs being used for parentage reconstruction (reviewed in Flanagan & Jones, 2019), where unknown or uncertain relationships are reconstructed using empirical data and software (e.g., Whalen, Gorjanc, & Hickey, 2019), and more complete pedigrees are used moving forward. Alternatively, there is an option to produce empirical estimates of relatedness for all founders or breeding individuals in conservation breeding programmes—as suggested in Ivy et al. (2016) and practised in Hogg et al. (2019)—and use this baseline of known relatedness moving forward using pedigrees. While the programme PMx allows for the inclusion of empirical data (Lacy et al., 2012), this approach requires caution, as the calculation of pedigree‐based identity by descent for subsequent generations—including kinship and gene diversity—will be affected by the addition of empirical data (Hogg et al., 2019). We acknowledge this approach requires further investigation and validation, particularly for species that receive periodic influx of wild individuals of unknown ancestry in their conservation breeding programme.

### 4.4 Future directions and concluding remarks

This study has produced pedigrees and whole‐genome sequences for two critically endangered species. Beyond estimating relatedness, these tools provide an exciting opportunity to explore other questions relevant to conservation, such as characterizing the genomic basis of fitness traits, including those associated with inbreeding depression (Kardos, Taylor, Ellegren, Luikart, & Allendorf, 2016; but see also Kardos & Shafer, 2019) or adaptation to captivity (e.g., Grueber et al., 2017). We also envision using the genomic resources developed here to further investigate best practice for making pairing recommendations, for example, agent‐based, multigenerational simulations can be used to evaluate whether genome‐wide diversity is best maximized using pedigrees, SNPs or a combination approach.

Given that SNPs have been successfully used to estimate relatedness for different purposes across a wide diversity of taxonomic groups outside of this study (as reviewed in Attard et al., 2018), we anticipate a SNP‐based approach for estimating relatedness and making subsequent pairing recommendations will be applicable beyond birds. In the meantime, for poorly pedigreed populations worldwide, we recommend a SNP‐based approach to estimate relatedness for subsequent pairing recommendations. It should be noted that many approaches used to date have used de novo reduced representation approaches (e.g., genotyping‐by‐sequencing, RADseq; Narum et al., 2013) for SNP discovery, which typically have more missing data, lower depth and fewer SNPs than the reference‐guided whole‐genome resequencing approach used here. While these factors may contribute to bias in relatedness estimates (but see Dodds et al., 2015), research still indicates that fewer SNPs, with more missing data and lower depth, than those presented here provide more precision than microsatellites (Attard et al., 2018). We expect reduced representation approaches will persist in the short term, especially for species with large and complex genomes (e.g., some fish, amphibians and invertebrates) that otherwise cannot yet be affordably resequenced across entire conservation breeding programmes. With that said, we also expect whole‐genome resequencing projects like ours will gain momentum in the years to come, as these data can be better leveraged to address multiple questions related to conservation genetic management (Harrisson, Pavlova, Telonis‐Scott, & Sunnucks, 2014; see also above). In the meantime, we look forward to seeing more poorly pedigreed conservation breeding programmes for taxonomically diverse species from around the world incorporate SNPs for estimating relatedness to inform pairing decisions.

## CONFLICT OF INTEREST

None declared.

## Data Availability

Genomic data provided in this manuscript are available through a password protected server on the Conservation, Systematics and Evolution Research Team's website (http://www.ucconsert.org/data/). Kakī and kākāriki karaka are taonga (treasured) species. For Māori (the indigenous people of Aotearoa), all genomic data obtained from taonga species have whakapapa (genealogy that includes people, plants and animals, mountains, rivers and winds) and are therefore taonga in their own right. Thus, these data are tapu (sacred) and tikanga (customary practices, protocols and ethics) determine how people interact with it. To this end, the passwords for the genomic data in this manuscript will be made available to researchers on the recommendation of the kaitiaki (guardians) for the iwi (tribes) that affiliate with kakī and kākāriki karaka.
