# Supplementary material for: A comparison of pedigree, genetic and genomic estimates of relatedness for informing pairing decisions in two critically endangered birds: Implications for conservation breeding programmes worldwide
Source: Evol Appl. 2020 Jan 27;13(5):991–1008. doi: 10.1111/eva.12916 (PMC7232769; doi:10.1111/eva.12916)
Supplement: Supplementary file 1 [file EVA-13-991-s001.docx]

Supplemental information for: A comparison of pedigree, genetic, and genomic estimates of relatedness for informing pairing decisions in two critically endangered birds: Implications for conservation breeding programmes worldwide.

Table of Contents

[S1.1: COANCESTRY Microsatellite Simulations 2](#_Toc25347068)

[S1.2: Compute Specifications for Genome Assembly 2](#_Toc25347069)

[S1.3: SNP Filtering 4](#_Toc25347070)

[S1.4: SNP-based relatedness estimates 7](#_Toc25347071)

[S1.5: MSI Correlations and MK Ranks 8](#_Toc25347072)

[S1.6: References 11](#_Toc25347073)

## S1.1: COANCESTRY Microsatellite Simulations

The programme COANCESTRY v. 1.0.1.9 (Wang et al., 2011) offers seven different estimators of relatedness for genetic and genomic markers, and to choose the most appropriate estimator for the kakī and kākāriki karaka microsatellite datasets, we employed the simulation module within COANCESTRY using allele frequencies, missing data, and error rates from our microsatellite datasets. To produce dyads that represent the relationships and degree of inbreeding found within kakī and kākāriki karaka, we used the R package ‘identity’ (Li, 2010) to generate 10,879 dyads for kakī and 1,484 dyads for kākāriki karaka  based on the pedigrees of both species. The frequency of each unique dyad in the kakī and kākāriki karaka datasets were scaled to create 1,000 dyads for each set that are representative of relationships between individuals used in this study. The COANCESTRY simulations were conducted using allele frequencies, error rates, and missing data rates from each microsatellite data set, with settings changed to account for inbreeding. The triadic likelihood approach (Wang, 2007) was selected given it had the highest Pearson’s correlation with ‘true’ relatedness and lowest variance for both kakī (Table S1) and kākāriki karaka (Table S2), as per Hammerly et al. (2013).

Table S 1: Average relatedness, variance, and correlation to ‘true’ relatedness for seven relatedness estimators using simulated datasets from kakī microsatellite allele frequencies and dyads in the kakī pedigree.

|  | TrioEst | West | LLEst | LREst | Rest | QGEst | MEst | Actual *R* |
| --- | --- | --- | --- | --- | --- | --- | --- | --- |
| Average | 0.214 | 0.063 | 0.064 | 0.067 | 0.065 | 0.064 | 0.236 | 0.07 |
| Variance | **0.047** | 0.131 | 0.125 | 0.088 | 0.098 | 0.103 | 0.051 | 0.023 |
| Pearson’s *r* | **0.511** | 0.407 | 0.418 | 0.483 | 0.473 | 0.451 | 0.502 | 1 |

Table S 2: Average relatedness, variance, and correlation to ‘true’ relatedness for seven relatedness estimators using simulated datasets from kākāriki karaka microsatellite allele frequencies and dyads in the kākāriki karaka pedigree.

|  | TrioEst | West | LLEst | LREst | Rest | QGEst | MEst | Actual *R* |
| --- | --- | --- | --- | --- | --- | --- | --- | --- |
| Average | 0.283 | 0.145 | 0.149 | 0.159 | 0.15 | 0.152 | 0.315 | 0.151 |
| Variance | **0.07** | 0.168 | 0.166 | 0.123 | 0.158 | 0.146 | 0.076 | 0.051 |
| Pearson’s *r* | **0.657** | 0.567 | 0.568 | 0.663 | 0.533 | 0.597 | 0.661 | 1 |

## S1.2: Compute Specifications for Genome Assembly

To assemble the kākāriki karaka reference genome, the following compute specifications were used: a 24 central processing unit (CPU) AMD Threadripper control unit (CU), 128 Gb RAM, 2.7 Tb Solid-State hard disk space were used. The run time for the assemblies was 30 hours for Meraculous, and about 121 hours for Masurca, per iteration. Several iterations were performed to compare assemblies with different parameters.

## S1.3: SNP Filtering

Base filtering was applied to all tried filtering datasets, including filtering to retain only biallelic SNPs with a minor allele frequency (MAF) greater than 0.05, a quality score greater than 20, and maximum missingness of 10% per site. This base filtering was designed to increase the quality, completeness, and reliability of SNPs by removing low quality markers and potential artefacts captured by rare multiallelic sites (Campbell et al., 2016) or low frequency sequencing error.

Different depth filters for each site were tested for each dataset to achieve an average of ~10x depth across all sites for each individual, to provide more certainty around reference-guided SNPs used here. Preliminary testing with kakī revealed that using an average minimum depth of 10x was sufficient for meeting this criteria (e.g., the lowest per individual average depth observed post filtering was 9.6x, with mean depth across all sites and individuals of 28.7x ± 10.29 SD). This depth was also used for an aligned study (see Galla et al. 2019). Applying this same filtering scheme to the kākāriki karaka dataset did not achieve an average depth of ~10x across all sites for each individual (the minimum average depth = 5.44x, mean depth across all sites and individuals = 15.93x ± 10.11). Therefore, a filtering trial using two different depth filtering strategies was employed, with one filtering using a hard minimum cut-off of 5x depth and the other using an average minimum depth of 20x. Both of these filtering trials also employed a hard maximum cutoff of 200x depth, to filter obvious high-coverage sites that come from collapsed repeats in the reference genome. Because there are known parent-offspring relationships represented in our dataset, and parent-offspring genomic contribution is 50%, we used relatedness between parents and offspring as a biologically meaningful measure to understand which approach best approximated 0.5 with the greatest precision. It was found that a hard minimum cut-off of 5x per SNP resulted in relatedness estimates that were more accurate and precise (Table S3).

Table S3: Results from filtering trials for depth, r^2^, and HWE using the kākāriki karaka data set. Base filtering refers to filter steps for biallelic SNPs with a MAF of 0.05, and missingness of 0.1. Scaled refers to the chosen data set, after scaling for self-relatedness to be equal to 1. Bold text refers to filtering scheme chosen. Alternating blue and white cell fill denotes trials with aligned depth and r^2^ settings.

| Trial | Average *R* ± SD | Average PO *R ±* SD | Min PO *R* | Max PO *R* |
| --- | --- | --- | --- | --- |
| Base filtering, minimum depth of 5x, maximum depth 200x, r^2^ 0.4 | 0.25 ± 0.1 | 0.43 ± 0.04 | 0.36 | 0.57 |
| Base filtering, minimum depth of 5x, maximum depth 200x, r^2^ 0.4, HWE | 0.02 ± 0.22 | 0.43 ± 0.11 | 0.19 | 0.67 |
| **Base filtering, minimum depth of 5x, maximum depth 200x, r^2^ 0.6** | **0.24 ± 0.10** | **0.43 ± 0.04** | **0.35** | **0.58** |
| **Base filtering, minimum depth of 5x, maximum depth 200x, r^2^ 0.6, Scaled** | **0.29 ± 0.12** | **0.53±0.03** | **0.47** | **0.67** |
| **Base filtering, minimum depth of 5x, maximum depth 200x, r^2^ 0.6, HWE** | **0.02 ± 0.22** | **0.43 ± 0.12** | **0.19** | **0.68** |
| Base filtering, minimum depth of 5x, maximum depth 200x, r^2^ 0.8 | 0.24 ± 0.11 | 0.44 ± 0.04 | 0.36 | 0.59 |
| Base filtering, minimum depth of 5x, maximum depth 200x, r^2^ 0.8, HWE | 0.02 ± 0.23 | 0.45 ± 0.12 | 0.21 | 0.7 |
| Base filtering, average minimum depth 20x, maxiumum depth 200x *r*^2^ 0.4 | 0.08 ± 0.13 | 0.33 ± 0.08 | 0.17 | 0.53 |
| Base filtering, average minimum depth 20x, maxiumum depth 200x *r*^2^ 0.4, HWE | 0.01 ± 0.19 | 0.36 ± 0.11 | 0.12 | 0.58 |
| Base filtering, average minimum depth 20x, maxiumum depth 200x *r*^2^ 0.6 | 0.05 ± 0.15 | 0.33 ± 0.09 | 0.14 | 0.55 |
| Base filtering, average minimum depth 20x, maxiumum depth 200x *r*^2^ 0.6, HWE | 0.01 ± 0.19 | 0.36 ± 0.12 | 0.12 | 0.59 |
| Base filtering, average minimum depth 20x, maxiumum depth 200x *r*^2^ 0.8 | 0.03 ± 0.17 | 0.34 ± 0.07 | 0.21 | 0.57 |
| Base filtering, average minimum depth 20x, maxiumum depth 200x *r*^2^ 0.8, HWE | 0.00 ± 0.20 | 0.37 ± 0.12 | 0.12 | 0.62 |

In addition to depth for kākāriki karaka, different *r*^2^ filters (i.e., *r*^2^ = 0.4, 0.6, and 0.8) for linkage disequilibrium were applied to see how this variable affected relatedness estimates. Further, HWE filters of 0.05 using KGD were applied to each of these trials to see if using a HWE filter on each of these approaches affected the accuracy and precision of relatedness (Tables S4 and S5). Overall, using a HWE filter resulted in less accurate and precise estimates of relatedness, which may be attributed to our datasets consisting mostly of family groups, thereby violating the assumption of random breeding. Strong to moderate LD filters (*r*^2^ = 0.4, 0.6) produced more accurate and precise estimates of relatedness in kakī, but did not make a substantial difference in kākāriki karaka. A moderate LD filter (*r*^2^ = 0.6) was chosen for both datasets (Tables S3 and S4).

Table S4: Results from filtering trials for r2 and HWE using the kakī data set. Base filtering refers to set filters for biallelic SNPs with a MAF of 0.05, an average mean depth of 10x, and missingness of 0.1. Scaled refers to the chosen data set, after scaling for self-relatedness to be equal to 1.

| Trial | Average *R* ± SD | Average PO *R* ± SD | Min PO *R* | Max PO *R* |
| --- | --- | --- | --- | --- |
| Base filter, *r*^2^ 0.4 | 0.33 ± 0.07 | 0.52 ± 0.03 | 0.47 | 0.60 |
| Base filter, *r*^2^ 0.4, HWE | 0.02 ± 0.12 | 0.37 ± 0.06 | 0.30 | 0.55 |
| **Base filter, *r*^2^ 0.6** | **0.25 ± 0.08** | **0.47 ± 0.04** | **0.42** | **0.59** |
| **Base filter, *r*^2^ 0.6, Scaled** | **0.27 ± 0.09** | **0.54 ± 0.03** | **0.49** | **0.61** |
| Base filter, *r*^2^ 0.6, HWE | 0.01 ± 0.13 | 0.38 ± 0.08 | 0.28 | 0.59 |
| Base filter, *r*^2^ 0.8 | 0.16 ± 0.09 | 0.42 ± 0.06 | 0.34 | 0.58 |
| Base filter, *r*^2^ 0.8, HWE | 0.00 ± 0.13 | 0.37 ± 0.09 | 0.26 | 0.60 |

## S1.4: SNP-based relatedness estimates

To produce pairwise estimates of relatedness using whole-genome SNPs, we used the R script KGD (Dodds et al. 2015), as it was designed to estimate relatedness using reduced-representation and resequence data while taking into account read depth. We also scaled our KGD relatedness values so that self-relatedness was equal to one, for two reasons: 1) Creating a diagonal with a value of 1 simplified Mantel tests performed in relatedness comparison analyses, and 2) Scaling KGD values created parent-offspring relatedness values that approximated 0.5 closer than unscaled KGD relatedness values (Tables S6). Because the inbreeding coefficient (*F*) can be derived from marker-based self relatedness (*R_self_*) where *F* = *R_self_* – 1 (Dodds et al. 2015), we anticipate that scaling may have accounted for variance in inbreeding values amongst sampled individuals. This scaling has shown to have minimal bias, as scaled and unscaled KGD relatedness values correlate with one another (Pearson’s *r* = 0.99, p < 0.001 for both kakī and kākāriki karaka, Table S7 and S8). Further, downstream MSI scores and MK ranks using KGD scaled and unscaled relatedness values are highly concordant with one another, indicating low bias from scaling (Figure S2).

In order to evaluate the performance of scaled KGD values, estimates of relatedness were compared to other marker-based relatedness estimators, including the triadic likelihood method (i.e., TrioML; Wang 2007), the *r_xy_* estimator (Hedrick & Lacy, 2015), and the KING estimator (Waples et al. 2019). The TrioML and *r_xy_* methods are particularly applicable to our study systems, as they account for inbreeding in their relatedness estimate. The R package *related* (Pew et al. 2015) was used to produce TrioML. Settings were set to account for inbreeding and calculate 95% confidence intervals with a bootstrap value of 100. The *r_xy_* and KING estimators were produced using the programme ngsRelateV2 and final VCFs for resequencing datasets (Hanghøj et al. 2019). Known parent/offspring dyads were used as a benchmark to evaluate precision for each approach, as parents/offspring relatedness should approximate 0.5 (Speed & Balding 2015).

Results indicate that the scaled KGD approach was able to produce estimates of parent-offspring relatedness that more closely approximated 0.5 than TrioML, *r_xy_*, and KING estimators (Table S3). While KGD estimates were more precise for parent-offspring relationships than these approaches, these estimates still significantly correlate with other estimators for both kakī (Pearson’s *r* = 0.87-0.96, *p* < 0.001) and kākāriki karaka (Pearson’s *r* = 0.91-0.96, *p* < 0.001l; see Tables S7 and S8).

Table S5: SNP-based estimates of relatedness using KGD and TrioML approaches in kakī and kākāriki karaka.

| Species | Estimator | Average *R* ± SD | Min. *R* | Max. *R* | Average Parent-Offspring *R* ± SD | Min. Parent-Offspring *R* | Max. Parent-Offspring *R* |
| --- | --- | --- | --- | --- | --- | --- | --- |
| Kakī | KGD | 0.25 ± 0.08 | 0.11 | 0.59 | 0.48 ± 0.04 | 0.42 | 0.59 |
|  | **KGD – Scaled** | **0.27 ± 0.09** | **0.13** | **0.61** | **0.54 ± 0.03** | **0.49** | **0.61** |
|  | TrioML | 0.06 ± 0.06 | 0 | 0.40 | 0.27 ± 0.06 | 0.17 | 0.40 |
|  | KING | 0.18 ± 0.04 | 0.06 | 0.32 | 0.29 ± 0.01 | 0.28 | 0.32 |
|  | r_xy_ | 0.09 ± 0.08 | 0 | 0.5 | 0.34 ± 0.07 | 0.23 | 0.50 |
| Kākāriki karaka | KGD | 0.24 ± 0.10 | 0.07 | 0.58 | 0.43 ± 0.03 | 0.35 | 0.58 |
|  | **KGD – Scaled** | **0.29 ± 0.12** | **0.08** | **0.67** | **0.53 ± 0.03** | **0.47** | **0.67** |
|  | TrioML | 0.07 ± 0.10 | 0 | 0.43 | 0.26 ± 0.04 | 0.18 | 0.43 |
|  | KING | 0.16 ± 0.07 | -0.06 | 0.33 | 0.28 ± 0.02 | 0.22 | 0.33 |
|  | r_xy_ | 0.09 ± 0.12 | 0 | 0.58 | 0.34 ± 0.05 | 0.25 | 0.54 |

Table S6: Pearson’s correlation coefficient between different SNP-based estimates of relatedness in kakī.

|  | KGD | KGD Scaled | TrioML | Rab | KING |
| --- | --- | --- | --- | --- | --- |
| KGD | 1 | — | — | — | — |
| KGD Scaled | **0.99** | 1 | — | — | — |
| TrioML | 0.96 | **0.96** | 1 | — | — |
| r_xy_ | 0.95 | **0.96** | 0.99 | 1 | — |
| KING | 0.80 | **0.87** | 0.85 | 0.86 | 1 |

Table S7: Pearson’s correlation coefficient between different SNP-based estimates of relatedness in kākāriki karaka.

|  | KGD | KGD Scaled | TrioML | Rab | KING |
| --- | --- | --- | --- | --- | --- |
| KGD | 1 | — | — | — | — |
| KGD Scaled | **0.99** | 1 | — | — | — |
| TrioML | 0.96 | **0.96** | 1 | — | — |
| r_xy_ | 0.96 | **0.96** | 0.99 | 1 | — |
| KING | 0.89 | **0.91** | 0.88 | 0.88 | 1 |

## S1.5: MSI and MK Rank Correlations

Pearson’s correlations between pedigree-, microsatellite-, and SNP-based MSI scores (Figure S1) and MK ranks (Figure S2) were performed in the manuscript. Scatterplots showing these relationshipsm abd associated Pearson’s *r* values are provided below.


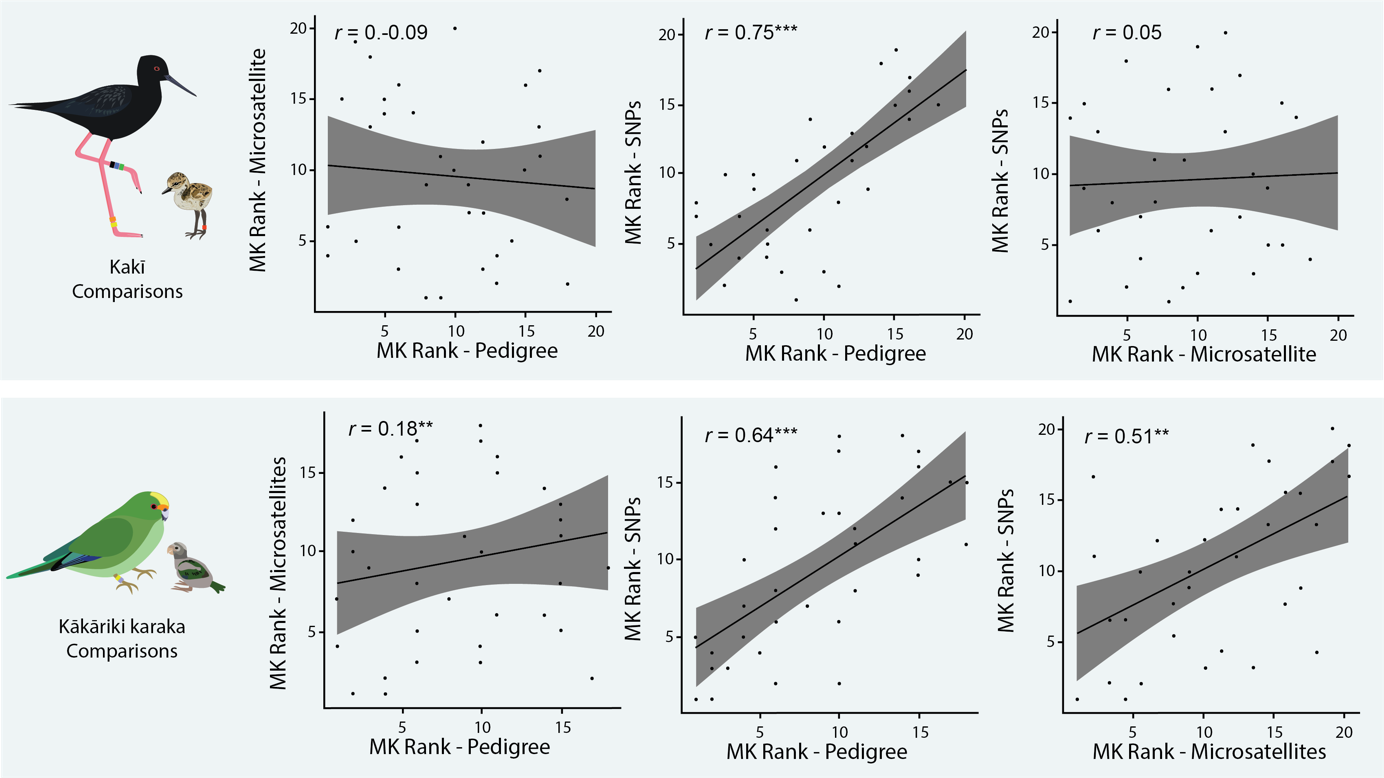


Figure S 1. Scatterplots showing relationships between pedigree-, microsatellite-, and SNP-based MSI scores in kakī and kākāriki karaka. Darker points denote higher frequencies than lighter points. A trend line (black) and 95% confidence intervals (grey) are shown in each comparison.


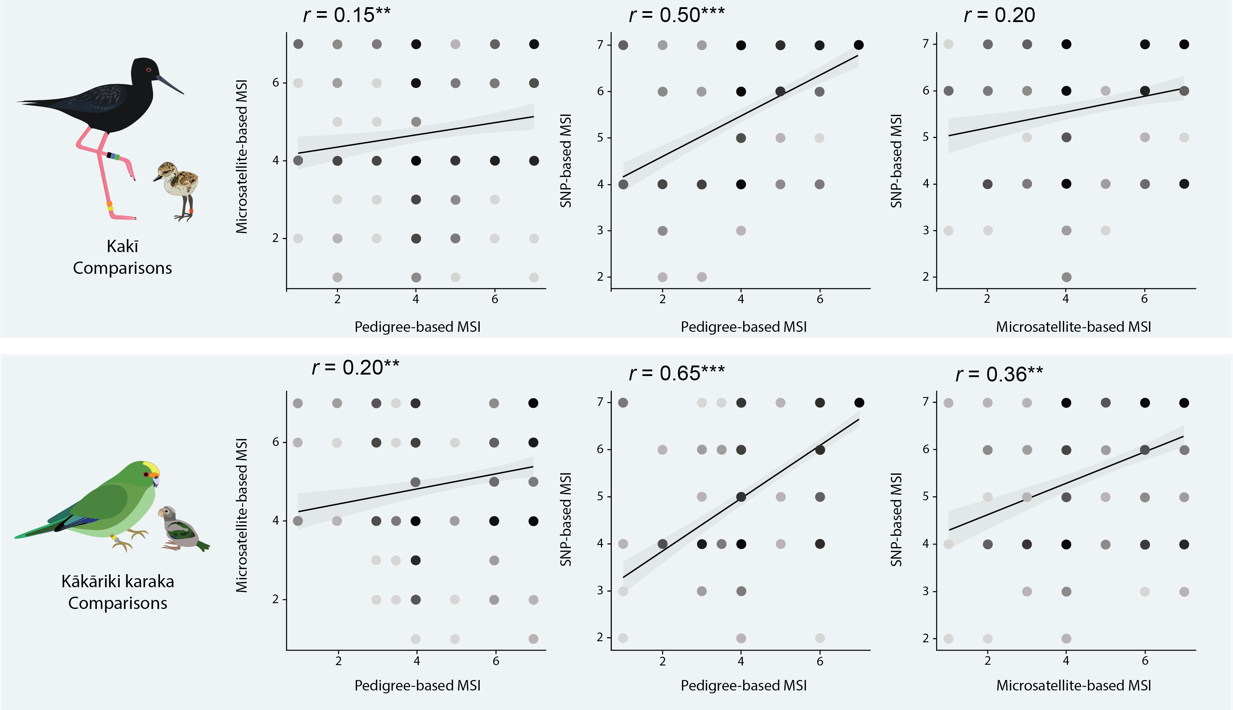


Figure S 2. Scatterplots showing relationships between pedigree-, microsatellite-, and SNP-based MK ranks in kakī and kākāriki karaka. Darker points denote higher frequencies than lighter points. A trend line (black) and 95% confidence intervals (grey) are shown in each comparison. Correlation coefficients are provided in the upper left hand corner of each graph, with ** indicating p < 0.01 and *** indicating p <0.001.

Pearson’s correlations were also used in the manuscript to understand the downstream effects of scaling on MSI scores and MK rank. Scatterplots showing these relationships are provided below (Figure S3).

Figure S 3. Scatterplots showing relationships between scaled and unscaled KGD estimates in regards to MSI scores and MK rank in kakī and kākāriki karaka. Darker points denote higher frequencies than lighter points. A trend line (black) and 95% confidence intervals (grey) are shown in each comparison.


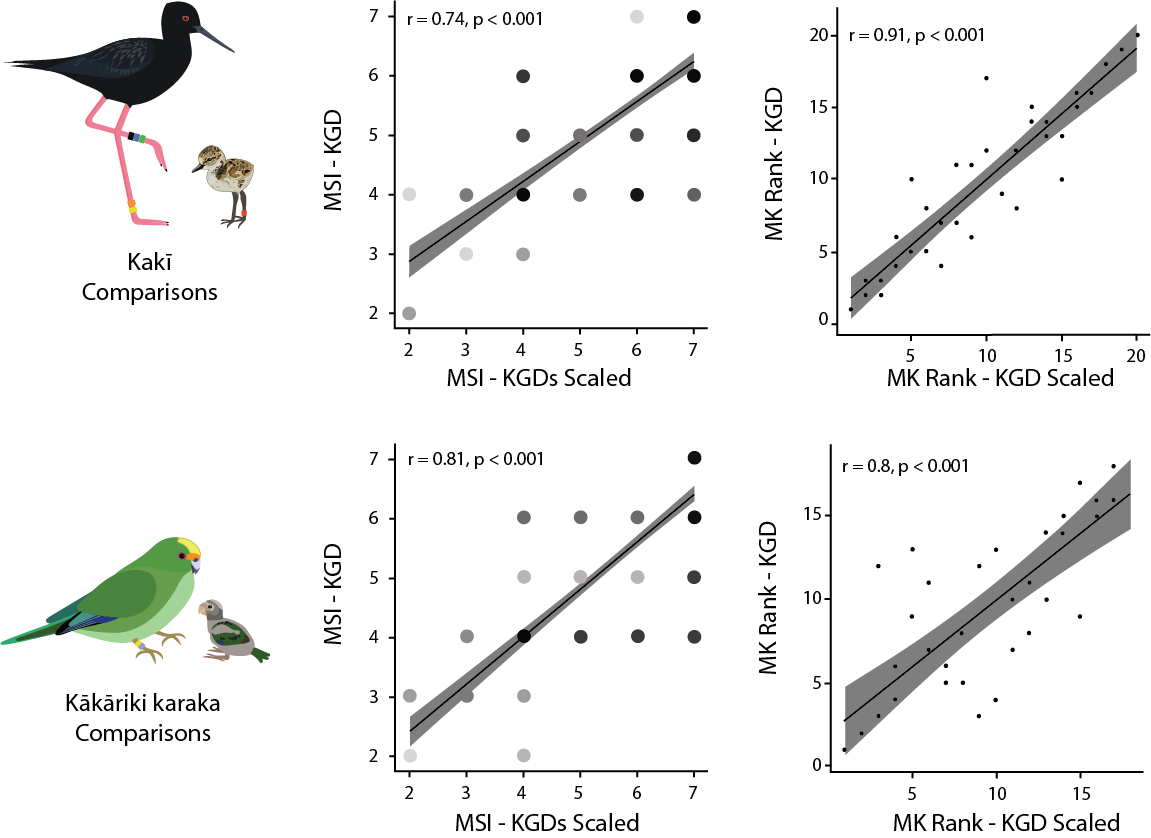


## S1.6: References

Campbell, I. M. , Gambin, T., Jhangiani, S., Grove, M. L., Veeraraghavan, N., Muzn, D. M., Shaw, C. A., Gibbs, R. A., Boerwinkle, E., Yu, F., Lupski, J. R. (2016). Multiallelic positions in the human genome: challenges for genetic analyses. *Human Mutations*, 37(3), 231-234.

Dodds, K. G., McEwan, J. C., Brauning, R., Anderson, R. M., van Stijn, T. C., Kristjánsson, T., Clarke, S. M. (2015). Construction of relatedness matrices using genotyping-by-sequencing data. *BMC Genomics*, *16*, 1047.

Hammerly, S. C., Morrow, M. E., Johnson, J. A. (2013). A comparison of pedigree- and DNA-based measures for identifying inbreeding depression in the critically endangered Attwater’s Prairie-chicken. *Molecular Ecology, 22*, 5313–5328.

Hanghøj, K., Moltke, I., Andersen, P. A., Manica, A., & Korneliussen, T. S. (2019). Fast and accurate relatedness estimation from high-throughput sequencing data in the presence of inbreeding. *GigaScience*, *8*(5), 1-9.

Hedrick, P. W., & Lacy, R. C. (2015). Measuring relatedness between inbred individuals. *Journal of Heredity*, *106*(1), 20-25.

Li, M. (2010). identity: Jacquard condensed coefficients of identity. R Package v.0.2-1.

Pew, J., Muir, P. H., Wang, J., Frasier, T. R. (2015). Related: an R package for analysing pairwise relatedness from codominant molecular markers. *Molecular Ecology Resources*, *15*, 557-561

Speed, D., Balding, D. J. (2015). Relatedness in the post-genomic era: is it still useful? *Nature Reviews Genetics*, 16, 33–44.

Wang, J. (2007). Triadic IBD coefficients and applications to estimating pairwise relatedness. *Genetics Research, 89,* 135-153.

Wang, J. (2011). COANCESTRY: A program for simulating, estimating and analysing relatedness and inbreeding coefficients. *Molecular Ecology Resources*, *11*, 141-145.

Waples, R. K., Albrechtsen, A., & Moltke, I. (2019). Allele frequency‐free inference of close familial relationships from genotypes or low‐depth sequencing data. *Molecular Ecology*, *28*(1), 35-48.
